# Land-use preferences of the European green toad (*Bufotes viridis*) in the city of Vienna (Austria): the importance of open land in urban environments

**DOI:** 10.1186/s12983-022-00480-x

**Published:** 2023-01-17

**Authors:** Lukas Landler, Stephan Burgstaller, Silke Schweiger

**Affiliations:** 1 Institute of Zoology, University of Natural Resources and Life Sciences (BOKU), Gregor-Mendel-Straße 33/I, 1180 Vienna, Austria; 2 First Zoological Department, Herpetological Collection, Natural History Museum Vienna, Burgring 7, 1010 Vienna, Austria

**Keywords:** Urban ecology, Amphibia, Anura, Landcover, Conservation

## Abstract

Urban areas are increasing worldwide, which poses threats to animal wildlife. However, in certain cases cities can provide refuges for endangered animals. The European green toad (*Bufotes viridis*) is one of such examples, which is known from cities throughout their distribution. In contrast, considerable areas of their former (primary) habitats have been degraded. The primary habitats of this species include steppes and wild river floodplains, both characterized by dynamic changes and the presence of open areas. We used available green toad observation data (2007–2020) to model the effects of land-use types on occurrence probability in the city of Vienna. Forest and densely populated areas were highly significantly negatively associated with green toad presence, while transformation/construction site areas showed a strong positive effect. Such occurrence pattern might be characteristic for early succession species, which depend on stochastic environmental disturbances (e.g., droughts and floods) in their primary habitats. We argue that urban landscape planning should appreciate the potential ecological value of open land in cities which is either in a transition phase or a permanent ‘wasteland’. Ecological managing of such landscape could vastly increase urban biodiversity.

## Background

Urban areas as well as human population densities in cities are increasing worldwide [1, 2]. Such developments are increasing floor sealing, reducing overall biodiversity and, in general, threatening many plant and animal wildlife species [3–6]. Often urban areas are dominated by relatively few urban exploiter species, which thrive under such conditions, taking advantage of food supply and reduced species competition or predation risk (i.e., rock pigeon, brown rat, house sparrow) [7, 8]. On the other hand, while in rural areas agricultural practices are intensifying and road networks are expanding, urban green spaces can provide refuge for threatened and otherwise rare animals [9, 10]. Van Helden et al. [11] found that, in South-West Australia western ringtail possums (*Pseudocheirus occidentalis*) can survive exclusively in residential gardens with no noticeable detrimental effects. In an expanding urban area in Ghana Ofori et al. [12] showed that urban green areas can conserve large proportions of the native small mammal biodiversity.

However, while some animal taxa, such as birds and small mammals, have several urban exploiters in their midst, others struggle considerably with anthropogenic transformations. Amphibians, for example, are under threat globally. It is estimated that currently over 40% of amphibian species are threatened or endangered [13]. Factors contributing to amphibian decline include fungal disease, pollution, land degradation as well as urbanization [14–16]. For many amphibian species, increased drainage and the resulting loss of natural water bodies leads to rapid population declines [17–19]. Amphibian species richness usually declines with increasing urbanization because anthropogenically altered environments often fail to provide suitable breeding sites as well as terrestrial habitats [15]. However, urban parks for example can provide an oasis for specialized amphibian species and contribute to amphibian conservation [16, 20]. For instance, the Sydney Olympic Park harbors the largest known population of the endangered green and golden bell frog (*Ranoidea aurea*) [21]. The European green toad (*Bufotes viridis*) is another amphibian species, that is well-known for the presence in urban areas [22–26], while their primary habitats (steppes and wild river floodplains) have been degraded in many areas, especially in central Europe. It has recently been suggested that green toads tend to continuously move towards the city centers in contrast to other amphibians, such as the common toad (*Bufo bufo*), which showed the opposite trend [25]. In general they are considered pioneer species inhabiting newly formed breeding sites very quickly [27]. Green toads are known to tolerate higher salt concentrations in their breeding habitats than other amphibians, enabling them to breed in salty steppe lakes and brackish water [28, 29]. Such tolerance, to salt and other pollutants, may help them to inhabit urban environments. However, a study in Germany indicated that anthropogenic land-use changes are reducing the green toads’ lifespan [30], hence, breeding success would need to counteract such effect in order to maintain stable populations [c.f., 31].

In Austria the green toad, with only few exceptions, occurs mainly in the East of the country, including remaining and former steppes and wild river floodplain areas [32]. In our study area in Vienna green toads have been reported from inner districts, as well as the suburbs [26, 33–35]. However, in-depth analysis of factors contributing to the occurrence of green toads in cities is still lacking, despite of numerous reports on urban populations. The habitat requirements we assume for green toads (preference for open and sunny terrain, and high tolerance for pollution) might be exemplary for a range of species that can seek refuge in city areas. Therefore, by identifying important landscape factors for the green toad, preserving such features will likely increase the overall biodiversity in cities.

To tackle our research question, we used the "Austrian Herpetofauna Database" of the Natural History Museum Vienna and open land-use data from the Viennese local government to model the presence and absence of green toads and thereby reveal their land-use dependences. While we expected that most urban land-use types have negative effects on green toad presence, we aimed to identify land-use types that positively affect green toad occurrence. This could be used to inform urban landscape planning and urban wildlife managing, especially with a focus on endangered species, that use urban areas as refuges.

## Methods

We used green toad occurrence data (from 2007 to 2020, n = 132 records) obtained from the "Austrian Herpetofauna Database" of the Natural History Museum Vienna. The observations recorded in the database originate from field surveys of the staff of the Herpetological Collection, data transferred by professional herpetologists and citizen scientists, scientific publications as well as unpublished investigations, like reports or degree theses, preserved collection specimens, historical indexes, and data exchange with organizations which store regional herpetological inventories. We did not differentiate between different type of reports or number of specimens found; every account of green toads was used as ‘presence’ in our analysis.

All analysis were done in the statistical software R [36]. To model toad presence, we used a generalized linear model with a binomial error distribution. Following the advice by Barbet-Massin et al. [37], we generated 10,000 pseudo-absences, which were randomly chosen from the area of Vienna, we adjusted the weights of the data points, in order for the weight sum to be identical between presences (n = 132) and pseudo-absences. Following the approach described in the dismo package [38] we extracted the sum of the land-use types surrounding each data-point in a radius of 500 m, after rasterizing the land-use data (using 25 m^2^ tiles). Land-use data (for 2012) was obtained from the Open Government Data platform [39]. We used the area of each land use type in a 500 m radius of each presence and pseudo-absence as predictors in our model, including all two-way interactions between land-use types. In order to avoid overfitting we performed an automated model selection using the buildmer package (function buildglmmTMB) [40], using least likelihood ratio tests. The model which performed best was then used to obtain model predictions (using ggpredict from the package ggeffects [41]). Model plots where obtained using the ggeffects plot function with adaptations using ggplot2 [42] functionality and stitched together using the package patchwork [43]. The model table was generated using the sjPlot package [44]. We plotted the land-use data using the ggplot function. The kernel density map of toad occurrence was created using the function ggmap [45] which uses a 2D kernel density estimation based on the kde2d function in the MASS package [46]. The background map of Vienna was downloaded using the function get_map, available from OpenStreetMap [47].

## Results

**Fig. 1:**
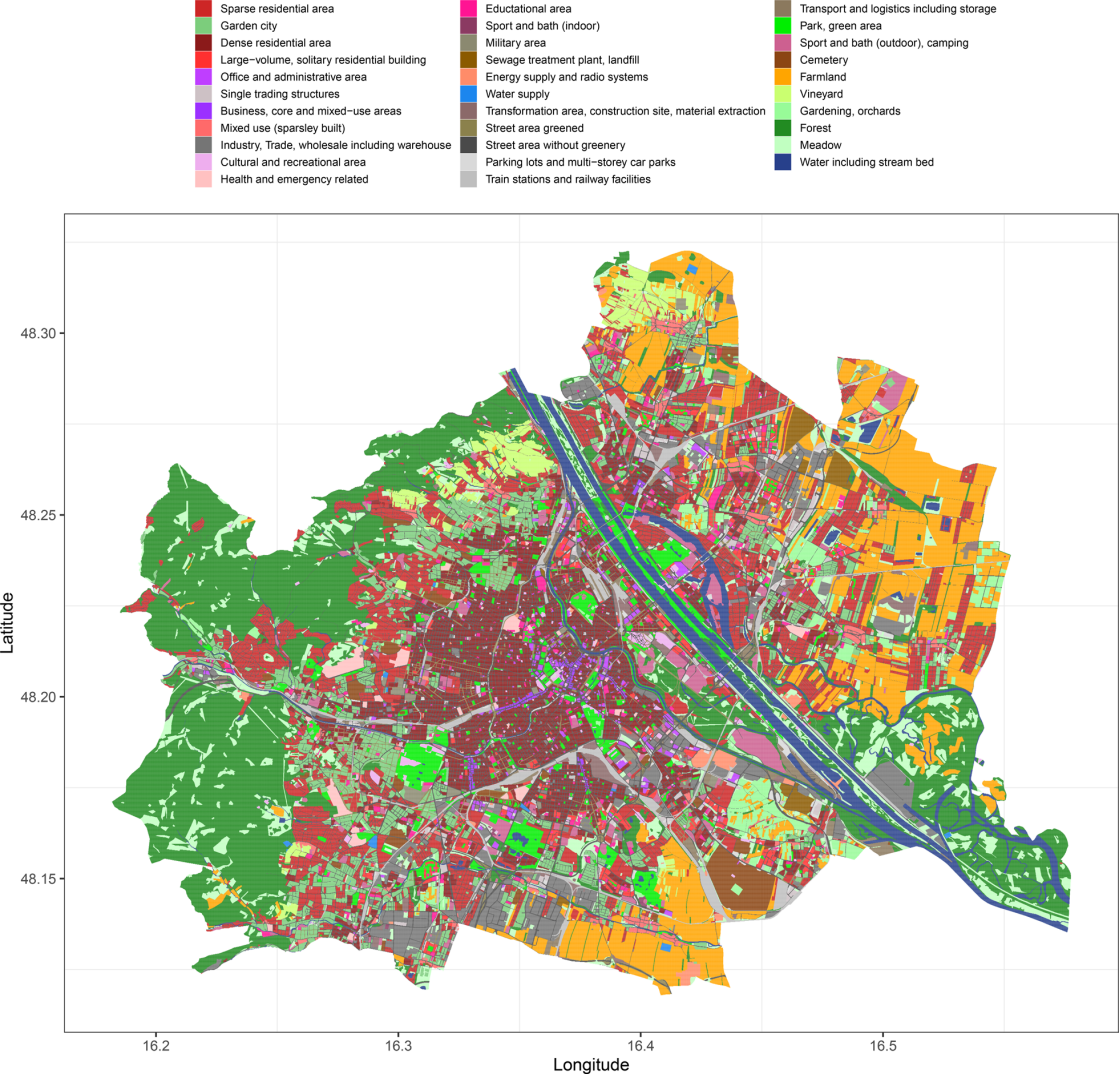
Land use types in Vienna based on the land-use data for 2012 provided by the City of Vienna through the Open Government Data platform [39]

**Fig. 2:**
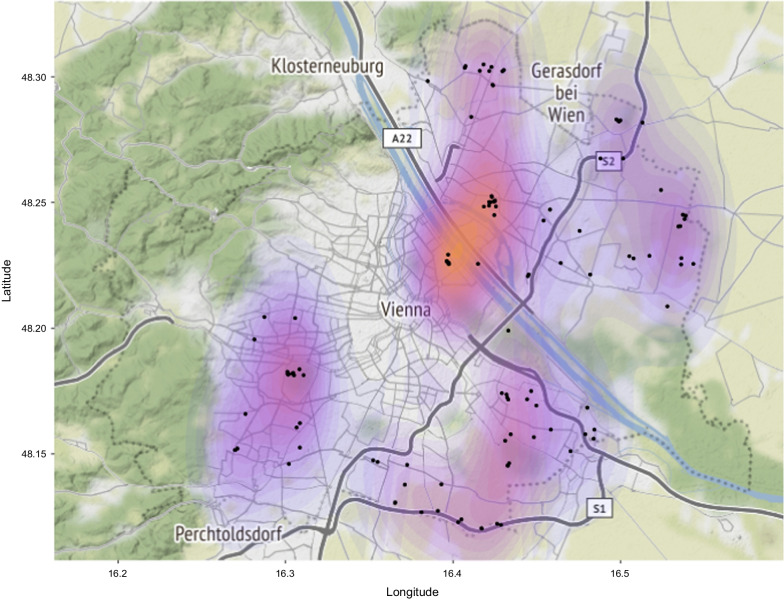
Kernel density representation of green toad occurrence in Vienna. Purple to orange scale corresponding to lower and higher occurrence rate of green toads based on the data base of the Natural History Museum Vienna (black dots represent toad records). The dotted grey line represents the city limits

**Fig. 3:**
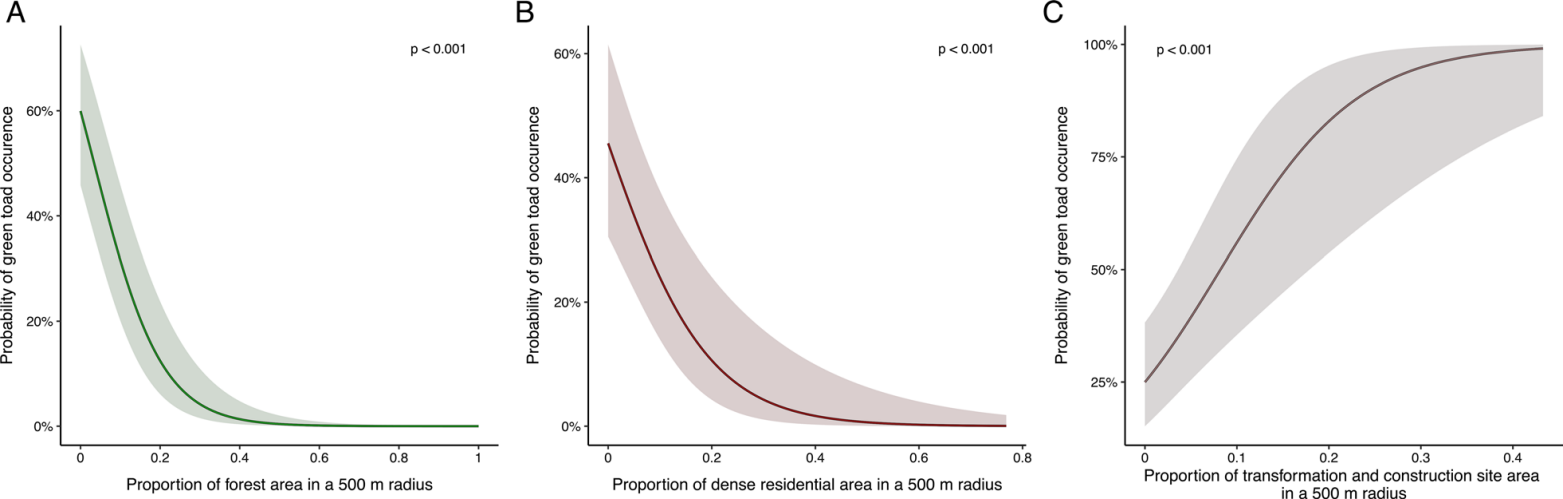
Model predictions of the three significant effects. P-values are shown in the plots. Forest and densely populated areas had strong negative impacts on toad occurrence while transformation/construction sites showed positive effects

Vienna’s land-use type distribution followed the expected pattern with more urban land-use types (e.g., densely populated housing areas) closer to the city core, while agricultural areas and forests were found more frequently farther away from the city center (Fig. 1). Green toads were recorded in most parts of the city except for the northwestern part (Fig. 2). After model selection nine land-use variables remained in the model (Table 1), the two land-use types with the strongest negative effects were forest and densely populated areas, only transformation/construction site areas were significantly positively associated with green toad occurrence (Fig. 3).

**Table 1:** Model results showing the effects of all included factors after model selection contributing to toad occurrence

Predictors	Green toad occurrence		
	Estimate	z	p
Forest	− 3.74 * 10^−04^	− 7.25	** < 0.001**
Dense residential area	− 3.11 * 10^−04^	− 3.80	** < 0.001**
Transformation area/construction site/material extraction	4.27 * 10^−04^	3.65	** < 0.001**
Gardening/orchards	1.28 * 10^−04^	1.60	0.111
Water supply	− 1.30 * 10^−02^	− 0.94	0.348
Industry/trade/wholesale including warehouse	− 1.57 * 10^−04^	− 1.39	0.166
Sparse residential area	− 9.01 * 10^−05^	− 0.80	0.421
Military area	4.25 * 10^−04^	1.73	0.083
Sport and baths/indoor sports facilities	− 6.75 * 10^−03^	− 1.27	0.203

Significant p-values (p < 0.05) are shown in bold

## Discussion

Despite the reported green toad occurrence in Vienna, and other cities, most factors analyzed contributed negatively to the green toad occurrence. Interestingly, the land-use parameter most negatively affecting green toad occurrence was ‘forest’. Forest areas exist in Vienna in several places and of various types, spanning from the woods in the western parts, which are a bit higher in elevation, to the low elevation floodplain related woods around the Danube. Such occurrence pattern is most likely related to the green toads’ preferences of open land (‘steppe species’[27]). The existing floodplain areas where green toads occur (for example around the river Tagliamento), are highly dynamic environments and include large open sand and gravel areas [48–50]. Such a strong negative impact of the land-use type ‘forest’, which is often considered ‘more natural’ or ‘less urban’, is important to consider when planning restoration efforts.

Green toads, as most animal species, were highly significantly negatively impacted by densely populated areas. Densely populated areas can act as a migration/movement barrier and lead to increased mortality due to roadkill and increased disturbance due to human activities [51, 52]. Mitigation measures should entail the reduction of traffic in the city, increasing green areas instead of pavement and whenever possible avoiding road construction. In addition, implementation of culverts was shown to reduce roadkill incidents of amphibians and other small animals [53, 54]. Given the lack of green toads in densely populated areas, open corridors between existing populations may allow animal movement and reduce the risk of inbreeding [55, 56].

Green toad occurrences were positively affected by the land use category “transformation areas and construction sites”, which is at least partly due to one major long-term construction area in the center of Vienna ("Nordbahnhofgelände", [57, 58]), with a well-known green toad population. However, it has been reported from other areas in Europe that green toads can appear quickly at new construction sites, using filled pits as breeding sites [22, 59]. In addition, our analysis, might underestimate the effects of this land use type as such sites are more dynamic than others and, for example, may not even be included to their full extent in the land use map we used. However, large construction sites are often planned many years in advance, leading to reduced or altered management of such transformation area. It could well be that these changes prior to construction are beneficial for the toads, not the building activity itself. Anyway, construction sites, similar to quarries [59–61], represent open habitats, without dense vegetation or sealed ground. In many ways, it does represent an early succession (i.e., ‘wasteland’) habitat type. However, as technology advances construction sites in cities will be available for shorter periods. In addition, increasing building activity usually increases ground sealing, and therefore, constitutes a loss of suitable habitats in the long run. Therefore, this would not be a sustainable conservation strategy for green toads (or any other species with such habitat needs). Instead, focusing on preserving such habitat type (i.e., ‘wastelands’ or fallows with early succession water bodies) could be a promising conservation strategy, however, this would involve regular maintenance work in order to prevent further habitat succession [62]. One might be surprised that we did not find green toads associated with the rivers (especially Danube) in Vienna, as rivers likely acted as the historic corridors for this species immigrating into central Europe. However, the contemporary urban rivers lack dynamic (i.e., floods and associated ephemeral ponds) and the protected areas along the rivers in Vienna are mainly forests, which are avoided by this species.

Our analysis, in accordance with published accounts on other species [21], shows that species which are not urban exploiters can find a niche in an otherwise dense urban fabric, if certain features are retained. Urban green areas are often dedicated to recuperation for humans and are therefore associated with planting trees, shrubs, flowering plants and in general cool and shaded recreational areas [63]. However, temporarily unmanaged open land, such as construction sites, fallows and ‘wastelands’, can provide essential refuge for endangered species and increase overall biodiversity in cities [64, 65]. The ecological value of such areas might be highly underappreciated, at least in the general public [66]. An interesting future avenue of investigation could focus on the community ecology of such ‘wasteland communities’ in cities in response to succession stage, surrounding land use, pollution and global warming (some of the topics already discussed in [67]). Such focus may elucidate the more dynamic aspects of urban wildlife in contrast to the typical urban park and woodland species assemblages. This may influence how policy makers treat and manage open areas (i.e., ‘wastelands’) and long-lasting construction sites in cities and provide a way to conserve rare species in cities.

## Data Availability

All data and materials will be made available upon request.
